# Nonketotic hyperglycemia with involuntary movements

**DOI:** 10.1590/0100-3984.2015.0253

**Published:** 2017

**Authors:** Tiago Medina Salata, Lívia de Oliveira Antunes, Bruno Niemeyer de Freitas Ribeiro, Rafael Silveira Borges, Diogo Goulart Corrêa

**Affiliations:** 1 Hospital Casa de Portugal, Rio de Janeiro, RJ, Brazil.; 2 Instituto Estadual do Cérebro Paulo Niemeyer, Rio de Janeiro, RJ, Brazil.

Dear Editor,

A 61-year-old woman who had been using insulin irregularly for the treatment of type II diabetes presented with hemichorea-hemiballism that had appeared suddenly in the left arm and left leg two weeks prior. Blood tests showed a blood glucose level of 450 mg/dL, a creatinine level of 0.9 mg/dL, and a urea level of 38 mg/dL. The complete blood count showed no abnormalities. The cerebrospinal fluid glucose concentration was 350 mg/dL. Magnetic resonance imaging (MRI) revealed a right-sided lesion, showing a hyperintense signal on T1-weighted images and a slightly hyperintense signal on T2-weighted images, located in the region of the caudate nuclei and putamen, with no enhancement, no evidence of bleeding in the magnetic susceptibility-weighted sequences, and no restricted diffusion on diffusion-weighted imaging (Figure 1). These imaging findings, together with the clinical and biochemical history, confirmed the diagnosis of hemichorea–hemiballism due to nonketotic hyperglycemia.

**Figure 1:**
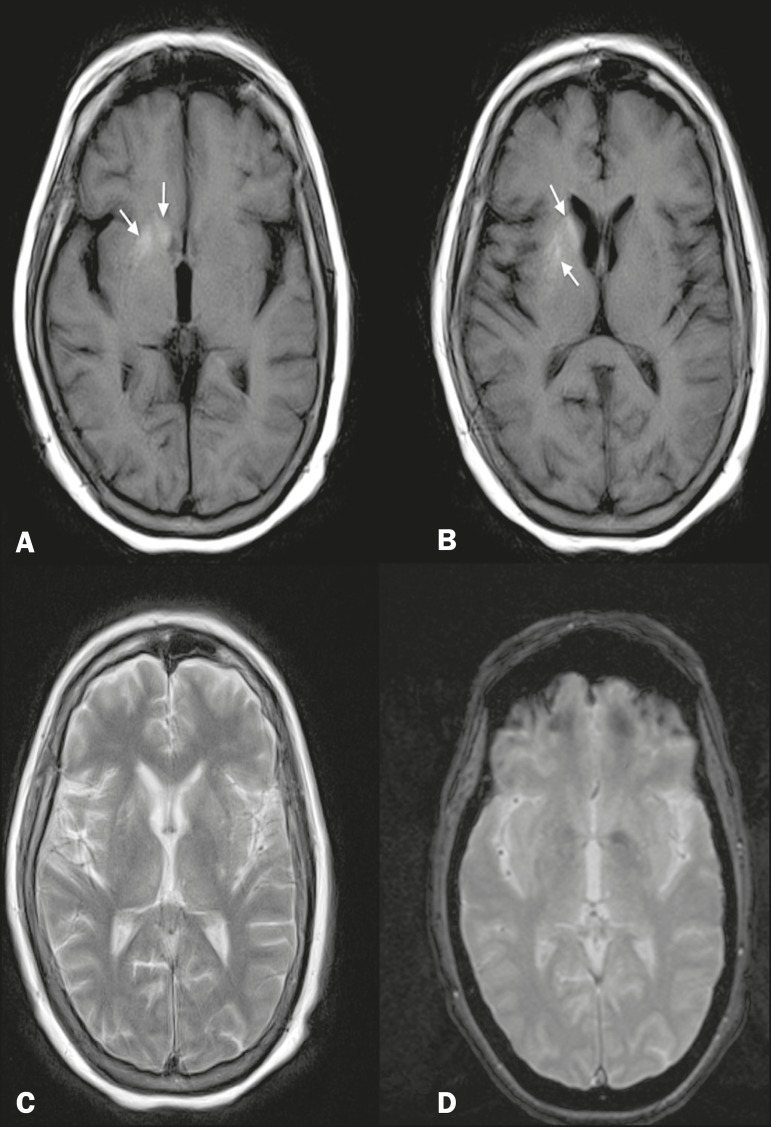
A,B: T1-weighted MRI showing a right-sided lesion with a hyperintense signal in the caudate nuclei and putamen (arrows). C: T2-weighted MRI showing a slightly hyperintense signal in the same regions. D: T2*-weighted MRI showing that there was no blood deposition in those regions.

Nonketotic hyperglycemia, also known as diabetic striatopathy, is a rare cause of involuntary movements as a primary manifestation of diabetes mellitus; it mainly affects elderly individuals, presenting as the triad of hemichorea-hemiballism, hyperglycemia, and a lesion in the basal nuclei showing a hyperintense signal on T1-weighted images^(1)^. Clinical and imaging findings are typically unilateral, although they can be bilateral in up to 11.4% of cases^(2)^, being potentially reversible and usually resolving within 2–12 months after the treatment of hyperglycemia^(3,4)^.

Although the pathophysiology of nonketotic hyperglycemia is unknown, potential mechanisms include metabolic changes such as the deposition of proteins and of degradation products of myelin, blood, calcium, or other minerals, which tend to decrease as serum glucose is controlled^(5)^. Another accepted theory is that a hyperglycemia-induced change in perfusion results in reduced Krebs cycle activity, inducing anaerobic metabolism, causing the brain to use alternative sources of energy, and metabolizing the gamma-aminobutyric acid (GABA) inhibitory neurotransmitter. In nonketotic hyperglycemia, GABA and acetate levels drop rapidly, leading to a decrease in acetylcholine synthesis. It has therefore been speculated that the reduced levels of acetylcholine and GABA in the basal nuclei leads to dysfunction of those nuclei, thus producing involuntary movements such as those seen in chorea-hemiballism^(6,7)^.

For the evaluation of central nervous system diseases, the imaging method of choice is MRI^(8-14)^. In hemichorea-hemiballism due to nonketotic hyperglycemia, MRI findings are characterized by lesions in the region of the caudate nucleus or lenticular nucleus, showing hyperintense signals in T1-weighted sequences and discretely hyperintense signals in T2-weighted sequences, without enhancement or diffusion restriction, such lesions typically being unilateral^(1,3,4)^, as in the case presented here. The diagnosis of lesions with high signal intensity in T1-weighted sequences of the region of the basal nuclei is broad; the following can be cited as some of the main causes^(1,4)^: hepatic encephalopathy; prolonged exposure to manganese; prolonged parenteral nutrition; Wilson’s disease; subacute intracerebral hemorrhage; exogenous carbon monoxide toxicity; and exogenous methanol toxicity. Correlation with the clinical and biochemical data is fundamental to making the definitive diagnosis^(1,4)^.

In conclusion, although the occurrence of hemichorea-hemiballism as a complication of uncontrolled diabetes is uncommon, the diagnosis should be considered when the clinical and MRI findings are characteristic of the disease. Thus, delays in the initiation of appropriate treatment can be avoided.

## References

[1] Bekiesinska-Figatowska M, Romaniuk-Doroszewska A, Banaszek M (2010). Lesions in basal ganglia in a patient with involuntary movements
as a first sign of diabetes - case report and review of the
literature. Pol J Radiol.

[2] Krishna S, Sodhi KS, Saxena AK (2015). Hyperdense basal ganglia in nonketotic
hyperglycemia. J Emerg Med.

[3] Bekiesinska-Figatowska M, Mierzewska H, Jurkiewicz E (2013). Basal ganglia lesions in children and adults. Eur J Radiol.

[4] Chokshi FH, Aygun N, Mullins ME (2014). Imaging of acquired metabolic and toxic disorders of the basal
ganglia. Semin Ultrasound CT MR.

[5] Hegde AN, Mohan S, Lath N (2011). Differential diagnosis for bilateral abnormalities of the basal
ganglia and thalamus. Radiographics.

[6] Aggarwal A, Bansal N, Aggarwal R (2016). Nonketotic hyperglycemia presenting as
monoballism. J Emerg Med.

[7] Hansford BG, Albert D, Yang E (2013). Classic neuroimaging findings of nonketotic hyperglycemia on
computed tomography and magnetic resonance imaging with absence of typical
movement disorder symptoms (hemichorea-hemiballism). J Radiol Case Rep.

[8] Machado VS, Silva Junior NA, Queiroz LS (2015). Central nervous system involvement in sarcoidosis. Radiol Bras.

[9] Dultra AHA, Noro F, Melo ASA (2015). Primary intercavernous lymphoma of the central nervous
system. Radiol Bras.

[10] Ribeiro BNF, Lima GA, Ventura N (2016). Chronic kernicterus: magnetic resonance imaging
findings. Radiol Bras.

[11] Langer FW, Suertegaray G, Santos D (2016). Hemichorea-hemiballism: the role of imaging in diagnosing an
unusual disorder in patients with nonketotic hyperglycemia. Radiol Bras.

[12] Ribeiro BNF, Salata TM, Borges RS (2016). Posterior reversible encephalopathy syndrome following
immunoglobulin therapy in a patient with Miller-Fisher
syndrome. Radiol Bras.

[13] Campos LG, Trindade RAR, Faistauer A (2016). Rhombencephalitis: pictorial essay. Radiol Bras.

[14] Georgeto SM, Zicarelli CAM, Gariba MA (2016). T1-weighted gradient-echo imaging, with and without inversion
recovery, in the identification of anatomical structures on the lateral
surface of the brain. Radiol Bras.

